# The Academy of the European Federation of Clinical Chemistry and Laboratory Medicine – benefits and opportunities

**DOI:** 10.1515/almed-2021-0033

**Published:** 2021-05-13

**Authors:** Ana-Maria Simundic

**Affiliations:** Department of Medical Laboratory Diagnostics, University Hospital “Sveti Duh”, Zagreb, Croatia; Faculty of Pharmacy and Biochemistry, University of Zagreb, Zagreb, Croatia; European Federation of Clinical Chemistry and Laboratory Medicine (EFLM), Milan, Italy; Task group EFLM Syllabus Course (TG-ESC), Milan, Italy

## The history of EFLM

The European Federation of Clinical Chemistry and Laboratory Medicine (EFLM) is a relatively young organization, but its roots go way back to the early 70s when first concerns were raised about the comparability of the profession between European countries, and this is when the idea of the regulation of the profession of Clinical Chemist was originally born. These early days of EFLM were nicely documented by several EFLM officers and this document, although never officially published in any journal, is the only written history of the EFLM [1]. EFLM was officially established in 2007 when the merger of The Forum of European Societies of Clinical Chemistry (FESCC) and the European Communities Confederation of Clinical Chemistry (EC4) occurred at the occasion of the General Meeting held at Euromedlab, in Amsterdam. EFLM was first named the European Federation of Clinical Chemistry (EFCC) and it was renamed into EFLM in 2013, by adding the Laboratory Medicine into its name, to better reflect the overarching character of EFLM, as the major organization in laboratory medicine in Europe.

Merger of FESCC and EC4 has created a true synergy, by joining values, competence, and activities of two organizations into EFLM. While FESCC was mainly active in promoting the education and scientific advancement of the profession, the care for the regulation of the profession in Europe was the core mission of the EC4. One of the key strategic goals of the EC4 was to achieve a Sectorial Directive for clinical chemists comparable to the Medical Directive, and it was clear that the way toward that goal is to produce an inventory of education and training in the various countries in EU at that time, and it was hoped that such developments would, in turn, open up the possibility to establish a registry of clinical chemists in Europe (the Register). This led to the publication of the first version of the European syllabus for post-graduate training in clinical chemistry and its several subsequent editions, of which the latest one has been published in 2018 [2], [3], [4], [5]. While the aim of the syllabus was to define the scope of education in clinical chemistry and areas in which a specialist in clinical chemistry should be able to demonstrate its knowledge, competence and skills, the idea of the Register was to ensure freedom of movement in the European Union, by setting the minimum entry standards of education and professional and managerial competence [6], [7], [8].

EFLM has, since its establishment, continued to operate along the path set by its predecessors. Although the final goal is not yet reached, one might say that we are possibly closer to it than ever. One of the key activities of EFLM over the past 14 years (from 2007–today) was to grow the Register and achieve recognition of Specialists in Laboratory Medicine, and thanks to these efforts the number of members of the Register has indeed substantially increased, but the true explosion in its size has occurred with the launch of the EFLM Academy in January 2020.

## The Academy

EFLM is an association of national societies in laboratory medicine from more than 40 countries in Europe, which represents >22,000 European specialists in laboratory medicine. The aims of EFLM are as follows:

– To promote and improve science and education within the clinical chemistry and laboratory medicine.
– To improve the efficiency, the quality and the safety of patient care through the highest standards of laboratory medicine.
– To represent clinical chemistry and laboratory medicine at European level vis-à-vis political, professional, scientific and other bodies, including patients’ organizations.
– To represent the professional interests of European specialists in clinical chemistry and laboratory medicine
– To promote the profession of specialist in clinical chemistry and laboratory medicine.
– To promote the certification and registration of clinical chemistry and laboratory medicine professionals through the EFLM Register of European Specialists in Clinical Chemistry and Laboratory Medicine.

To fulfill its aims and to help further grow the Register of European specialists in laboratory medicine, the EFLM has established the EFLM Academy. The official launch of the EFLM Academy was on January 1st 2020. The EFLM Academy is unique, exclusive on-line resource and communication platform through which EFLM aims to support the education, training and continuous professional development of laboratory medicine practitioners in Europe. As a leading professional entity in laboratory medicine in Europe, the EFLM Academy aims to serve as an educational resource not only to Specialists in laboratory medicine, but also to all others interested in laboratory medicine.

## Who is eligible to join the Academy?

EFLM Academy offers the individual type of membership. Eligible are all individuals who are interested in Laboratory Medicine. Representatives of diagnostic companies are also welcome to join the EFLM Academy.

## How can I join the EFLM Academy?

There are basically two distinct routes to join the EFLM Academy: i) through the block enrollment of National Societies and ii) by individual registration. Block enrollment is a preferred route, not only because it is more convenient for individual members of the national society, but also because it offers more benefits than the individual membership type.

Block enrollment is achieved through two simple steps:

1. The agreement between the EFLM and National society

The block enrollment is based on an agreement signed between EFLM and the National Society. All National societies interested to join the EFLM Academy through block enrollment are invited to contact the EFLM Office (eflm@eflm.eu) to receive the proposal of the agreement. It is important to note that the agreements signed during the current year will be valid for registrations starting in the coming year (i.e. if an agreement is signed in June 2021, registrations will start from Jan 1, 2022).

2. The list of members which will join the EFLM Academy

Once the agreement is signed between the EFLM and the National society, National society will be asked by the EFLM Office to submit the list of members who will become the members of the Academy in the coming year. This list has to be submitted by the end of the year (within 31st December, the latest).

A membership year runs from 1 Jan to 31 Dec of the calendar year. Since the above procedure may take a couple of months, it is recommended to start with it in the first half of the year, to allow enough time for its completion.

The individual membership is an alternative for all those coming from the societies which for whatever reason do not support the block enrollment. Applications are submitted on-line at the link: https://www.eflm.eu/academy-register/login. As already noted above, members who have joined through the individual registration may not enjoy a full package of benefits compared to those who have joined through the block enrollment.

## Academy vs. Register

While the Academy is open to all who are interested in Laboratory Medicine, those individuals who also meet the EFLM Equivalence of Standards (EoS) in education and training can join the EuSpLM Register. The applicants who are interested to join the Register (individual or a National society) shall be requested to provide the evidence that they meet EoS in education and training. This evidence is provided by the National society in the case of the block enrollment. In case of an individual application, the evidence shall be sought from an individual. For more details about the Register please visit EFLM website, under EFLM Register of Specialists in Laboratory Medicine (EuSpLM) (https://www.eflm.eu/site/page/a/1305).

The applications to the Register are evaluated by the EFLM Committee for Profession (C-P) and once the EoS is approved, those who meet the EoS criteria will be automatically enrolled in EFLM Register and shall be recognized as EuSpLM.

**Figure 1:**
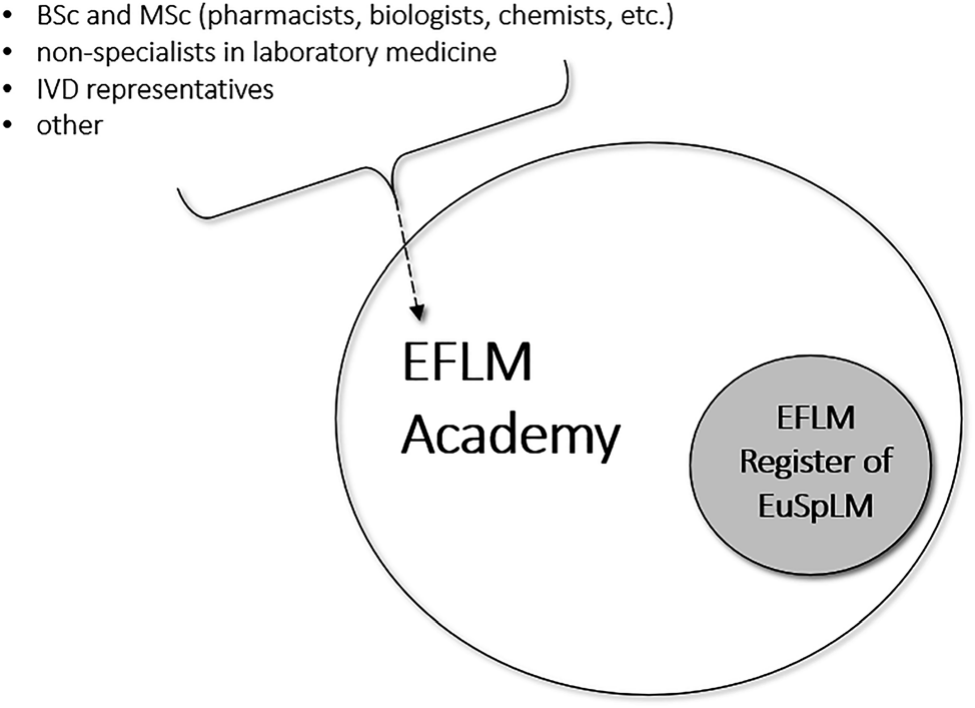
The relationship of the EFLM Academy and the EFLM Register.

The applications to the Register are possible only through the application to the Academy. The relationship between the Register and the Academy is described in Figure 1.

## The benefits for the members of the EFLM Academy

The package of benefits of the EFLM Academy has been carefully tailored to meet the needs of the laboratory medicine professionals in Europe. In particular, the benefits of the membership in the EFLM Academy, for the current year (2021) are listed below:

– Free on-line subscription to CCLM, the official EFLM journal,
– Free access to documents of the Clinical Laboratory Standards Institute (CLSI) (this applies only to EFLM Academy Members enrolled collectively through their National Society, by block subscription),
– CLSI membership at a reduced rate (25% of discount)-this applies to individual EFLM academy members,
– Reduced registration fee to all EFLM conferences and courses (this applies to EFLM Preanalytical conferences, EFLM Strategic Conferences, CELME Symposium, but does not apply to congresses/conferences organized in collaboration with other organizations, such as EuroMedLab),
– Free access to EFLM webinars,
– Regular e-mail notifications of all EFLM activities, programs, and opportunities.

In addition, only for members from EFLM National Societies/Associations:

– Eligibility to apply for EFLM travel grants,
– Enrollment in the EuSpLM Register for those who meet EFLM EoS in education and training.

The EFLM is committed to grow the package of benefits and keep offering new opportunities to its members in the future. While the above listed benefits are currently available (in 2021), it is to be pointed out that the Executive Board of EFLM has recently decided to substantially extend the Academy benefits by providing some new exciting opportunities, like the free access to the EFLM Syllabus course—the unique educational on-line resource covering the entire scope of laboratory medicine (available from January 2022), the free on-line subscription to several other laboratory medicine journals, more e-learning opportunities and by offering the opportunity to compete for some new awards (to be introduced in 2022), to name just a few.

It is hoped that the EFLM Academy will grow in its size in the future. Its value is demonstrated not only through providing support in education and training but also through catalyzing the recognition of European Specialists in Laboratory Medicine.

The higher purpose of being a member of the EFLM Academy, beyond enjoying its benefits, is to contribute to these goals, to contribute to the harmonization of the education and training in laboratory medicine as well as to contribute to the EFLM efforts aimed toward the recognition of European Specialists in Laboratory Medicine.

To be a member is not only a matter of choice. It may even be looked at as our professional responsibility. All are invited to join. We are stronger together.
